# News and Notes

**Published:** 1907-11

**Authors:** 


### NEWS AND NOTES.

---

The International Congress on Tuberculosis will meet in Washington, September 21st to October 12th, 1908.

---

The Southern Surgical and Gynecological Association will hold its next annual meeting at New Orleans, December 17, 18, and 19, 1907, under the presidency of Dr. Howard A. Kelly, of Baltimore. The secretary is Dr. William D. Haggard, of Nashville, who should be addressed concerning places on the program.

---

It is stated that 55,759 persons died in England and Wales from tuberculosis during the year 1905.

---

It is claimed that Manila is the cleanest city in the entire Orient.

---

There were 260 prisoners in the Court of Special Sessions of New York in one day, all charged with violations of the milk laws.

The committee on the Mary Putnam Jacobi fellowship announces that $8,000 of the $25,000 required has already been raised. The fund is expected to provide an income of $1,000, whereby efficient aid may be rendered to post-graduate women students in medicine.

---

The physicians and druggists of Augusta recently held a joint meeting of their respective societies and discussed the question of "counter-prescribing." A committee was appointed to consider the matter further.

---

The College of Physicians and Surgeons and the Atlanta School of Medicine have both begun their fall terms with an unusually large attendance.

Dr. G. Lane Tanneyhill, Baltimore, has been elected surgeon-general of the Grand Army of the Republic.

---

The State Board of Health of California is urging all citizens to aid in exterminating rats on account of the part they play in disseminating certain infectious diseases.

---

The physicians of Trenton have united to request the newspapers to refrain from mentioning their names in connection with cases which they may be treating.

---

The medical association of the district of Palermo, in Italy, have taken the initiative in refusing their services to duelists. They think that the inability to obtain a physician may help to deter would-be duelists, and arouse an agitation against dueling in general.

---

Six physicians of Norfolk, who failed to comply with the law which requires all physicians to report births to the board of health, have been summoned to appear in the police court to answer this charge.

---

The U. S. Consul at San Jose, Costa Rica, reports that owing to the prevalence of yellow fever in Cuba the government of Costa Rica has declared quarantine against that island.

The United States Civil Service Commission announces an examination on October 23, 24, 1907, to secure eligibles from which to make certification to fill a vacancy in the position of anatomist (male,) at $1,600 per annum, in the Army Medical Museum office of the Surgeon-General and other similar vacancies as they may occur there.

---

The governor has selected the following individuals for beneficiary scholarships in the Medical Department of the University of Georgia for the term of 1907-8:

State at Large—Sheddie, Usher, Springfield; W. K. Smith, Pembroke; William Massey, Barwick; F. L. Lanier, Sylvania; Clarence Cox, Ellijay; D. L. Deal, Statesboro.

First District—W. H. Sutton, Swainsboro; T. B. Brantley, Sylvania.

Second District—W. D. Sloan, Milltown, J. G. Standifer, Blakely.

Third District—G. G. Lunsford, Americus; J. W. Polhill, Hawkinsville.

Fourth District—Albert Martin, Columbus.

Fifth District—Mason Smith, Douglasville; T. R. Aycock, Monroe.

Sixth District—T. F. Bradley, Bradley; G. L. Johnson, Yatesville.

Eighth District—DeWitt Payne, Fort Lamar; Fred Griffith, Eatonton.

Ninth District—Ralph Freeman, Dacula; M. B. Ketron, Clarkesville.

Tenth District—H. N. Bussey, Thomson; John A. Johnston, Augusta.

Eleventh District—W. E. Williams, Soperton; D. W. F. Maloy, Beach.

---

The McGill University has planned a new building for its Medical School, the construction of which will begin at an early date. The cost is estimated at $500,000.00 and the Medical Department has established a five year course, the fifth year to be devoted entirely to hospital work.

